# Development and validation a methodology model for traditional Chinese medicine good practice recommendation: an exploratory sequential mixed methods study

**DOI:** 10.3389/fphar.2025.1501634

**Published:** 2025-01-31

**Authors:** Su Li, Luan Zhang, Yangyang Wang, Runsheng Xie, Wenjia Chen, Myeong Soo Lee, Yasser Sami Amer, Amin Sharifan, Heba Hussein, Hui Li

**Affiliations:** ^1^ The Second Clinical College, Guangzhou University of Chinese Medicine, Guangzhou, Guangdong, China; ^2^ Guangzhou University of Chinese Medicine, Guangzhou, Guangdong, China; ^3^ Guangdong Provincial Hospital of Chinese Medicine, Guangzhou, China; ^4^ The First Affiliated Hospital of Guangzhou University of Chinese Medicine, Guangzhou, Guangdong, China; ^5^ KM Science Research Division, Korea Institute of Oriental Medicine (KIOM), Daejeon, Republic of Korea; ^6^ Department of Pediatrics, King Saud University Medical City, Riyadh, Saudi Arabia; ^7^ Research Chair for Evidence-Based Health Care and Knowledge Translation, Family and Community Medicine Department, College of Medicine, King Saud University, Riyadh, Saudi Arabia; ^8^ Department of Internal Medicine, Ribeirão Preto Medical School, University of São Paulo, Ribeirão Preto, Brazil; ^9^ Department for Evidence-Based Medicine and Evaluation, University for Continuing Education Krems, Krems an der Donau, Lower Austria, Austria; ^10^ Oral Medicine, Oral Diagnosis, and Periodontology Department, Faculty of Dentistry, Cairo University, Cairo, Egypt; ^11^ State Key Laboratory of Traditional Chinese Medicine Syndrome, Research Group of Standardization of Chinese Medicine, The Second Affiliated Hospital of Guangzhou University of Chinese Medicine, Guangzhou, China

**Keywords:** traditional chinese medicine (TCM), guidelines, good practice recommendation (GPR), methodology model, mixed method

## Abstract

**Background:**

To develop a rational and standardized traditional Chinese medicine (TCM) good practice recommendation (GPR) methodology model that guides the formulation of recommendations grounded in clinical experience.

**Methods:**

We adopted an exploratory sequential mixed-method to develop a methodology model by coding systematically collected literature on methodology and TCM guidelines related to TCM GPR using a best-fit framework synthesis. Then based on real-world data (published TCM guidelines), saturation tests, structural rationality validation, and discriminability tests were conducted to validate methodology model.

**Results:**

A total of 35 methodological literature and 190 TCM guidelines were included. A TCM GPR methodology model was developed, including 3 themes, 10 sub-themes, and the relationships between themes and subthemes. The information of TCM GPR methodology model achieved data saturation. The fit indices were within the acceptable range, and were able to distinguish the overall differences between guidelines from different literature sources, development organizations, guideline types, discipline categories, and funding categories.

**Conclusion:**

The study developed a TCM GPR methodology model which describes the definition of a TCM GPR, how to formulate it, and how to report it. The methodology modeldemonstrates good fit, discriminability, and data saturation. It can standardize the specific formulation of TCM GPRs, facilitate the scientific and rational formation of TCM GPRs, and provide theoretical and methodological guidance for the formation of TCM GPRs.

## 1 Introduction

In 1992, the concept of evidence-based medicine was introduced and rapidly spread (Guyatt et al., 1992; Djulbegovic and Guyatt, 2017). Currently, the formulation of recommendations in traditional Chinese medicine (TCM) guidelines emphasizes evidence from systematic reviews (Verwoerd et al., 2021). However, when the evidence is insufficient, recommendations and guidance are still needed for some important and urgent questions (Ge et al., 2022; Liu et al., 2021). In response to such situations, guideline developers tend to use good practice approaches to introduce clinical experience, one of the three elements of evidence-based medicine, into guidelines to address the issues (Diekemper et al., 2018; Vermeulen et al., 2019; Guyatt et al., 2015; Guyatt et al., 2016; Dewidar et al., 2023).

Currently, there are two main research approaches in the field of TCM for incorporating clinical experience into clinical practice guidelines: The first approach is to integrate clinical experience into the evidence system, constructing a TCM-specific evidence body and subsequently developing recommendations based on this evidence system (Wang et al., 2013; Liu, 2007; Chen et al., 2019). The second approach involves translating clinical experience into recommendations outside the evidence system (Chen et al., 2013; Guo et al., 2008; Chang et al., 2022). These approaches represent active explorations for incorporating clinical experience into TCM guidelines. However, it is important to note that expert clinical experience should not be considered a research design and should not be included within the evidence system (Guyatt et al., 2008; Balshem et al., 2011; Oxman et al., 2006). In addition, the methods that operate outside of the evidence system lack systematic and standardized procedures, which undermines the quality of the recommendations and prevents alignment with recognized guideline methodologies.

In light of the issues discussed above, it is necessary to establish a systematic and standardized methodological system to standardize the formation of recommendations based on expert clinical experience in TCM guidelines. This study adopts an exploratory sequential mixed-methods approach to develop a TCM Good Practice Recommendation (GPR) methodology model, providing theoretical and methodological support for the formation of TCM GPR.

## 2 Methods

We adopted a mixed-methods approach grounded in an exploratory sequential design, where qualitative research based on best-fit framework synthesis was used to develop the TCM GPR methodology model (Carroll et al., 2011; Carroll et al., 2013). Then, quantitative research was conducted to validate this new model. Subsequently, an expert consensus meeting was held to review the results.

### 2.1 Systematically identify research samples

In our study, methodological literature on TCM GPR methods and TCM guidelines that mentioned TCM GPR methods were used as research samples. A comprehensive search was conducted using PubMed, EMBASE, Web of Science, China Biology Medicine Database (CBM), China National Knowledge Infrastructure (CNKI), Chinese Scientific Journal Database (VIP), and the Wanfang Database to search for methodological literature and TCM guidelines. Additionally, searches were performed on the China Association of Chinese Medicine (CACM) and Medlive (http://www.medlive.cn) websites for relevant TCM guidelines. Gray literature was also searched using Google Scholar. All relevant articles from their inception to February2023 were retrieved. The search strategy is provided in Supplementary Material 1.

#### 2.1.1 Inclusion criteria for methodological literature

Inclusion Criteria: Studies related to methods or theories that provide recommendations based on clinical experience when there is a lack of evidence during the development of TCM guidelines.

Exclusion Criteria: (1) Duplicated literature; (2) Unavailability of full-text electronic version; (3) Conference abstracts.

#### 2.1.2 Inclusion criteria for TCM guidelines

Inclusion Criteria: Including recommendations or opinions for clinical guidance based on clinical experience when there is a lack of evidence.

Exclusion Criteria: (1) Duplicated published literature; (2) Unavailability of electronic full text; (3) Interpretation, and evaluation of guidelines.

Two researchers independently screened the full-text articles. The results were then discussed with the research team to reach a consensus on the final inclusion of the methodological literature and TCM guidelines. SPSS 17.0 software was used to randomly select half of the TCM guidelines as coding guidelines for developing the methodology model. The remaining half of the TCM guidelines were used as testing guidelines to evaluate the saturation of the methodology model.

### 2.2 Model development

#### 2.2.1 Thematic analysis to generate a priori framework

Using the logical framework method, we began with the problem of TCM GPR as the core issue and gradually extrapolated downward, constructing a problem tree, which was then transformed into the corresponding initial analytical framework based on the causal relationships illustrated in the problem tree.

According to the initial analytical framework, the thematic analysis (Thomas and Harden, 2008) method was used to code the methodology literature line-by-line. The codes were then categorized and grouped to form an *a priori* framework with a three-tier structure of themes, subthemes, and categories. Two researchers independently coded and cross-checked codes, and inconsistencies were discussed and agreed upon.

#### 2.2.2 Synthesizing TCM GPR methodology model

Based on the *a priori* framework, we employed a line-by-line coding format to code the relevant information from the TCM guidelines into the corresponding themes/subthemes/categories in the priori framework. Information that could not be coded into the *a priori* framework was subjected to secondary thematic analysis to form new themes/subthemes/categories.

Ultimately, by merging the *a priori* frameworks with new themes and exploring potential linkages between the themes at each level, the TCM GPR methodology model was developed.

#### 2.2.3 Dissonance exploration

Dissonance exploration aims to identify any differences between the *a priori* framework and the TCM GPR methodology model.

### 2.3 Model validation

#### 2.3.1 Saturation test

A thematic saturation test was conducted using a theme saturation table. If five testing guidelines in succession do not yield any new themes, this indicates that the study has reached theme saturation (Yang et al., 2022; Constantinou et al., 2017).

#### 2.3.2 Structural rationality validation

SPSS 17.0 and AMOS 21.0 software were used to assess the reliability and validity of the methodology model. Three themes were treated as latent variables, while ten subthemes were considered as observed variables. A matrix coding table was generated for specific data analysis, with each subtheme of each guideline serving as a reference point.

Reliability: The reliability of the model was assessed using Cronbach’s α. Cronbach’s α ≥0.6 was considered indicative of good reliability (Pires et al., 2022).

Validity: Several indices were used to evaluate the goodness of fit, including χ^2^/df, root mean square error of approximation (RMSEA), goodness of fit index (GFI), and adjusted goodness of fit index (AGFI). For χ^2^/df <3 was considered a good fit. For RMSEA <0.1 was considered an acceptable model fit (Pires et al., 2022; Yan et al., 2023). For GFI >0.90 or AGFI >0.8 was considered a good fit (Lopez et al., 2023; Marsh et al., 1988).

Composite reliability (CR) and average variance extracted (AVE) were used to assess the convergent validity and discriminant validity. For CR >0.60 and AVE >0.5 was considered a good convergent validity (Zhou et al., 2022).

#### 2.3.3 Discriminability test

##### 2.3.3.1 TCM GPR evaluation

After discussions and consensus among the research group, the TCM GPR evaluation was based on the three themes of the methodology model as domains and nine sub-themes as items. The sub-theme “1.1 Main types” was excluded. Points were assigned to the initial codes under the items, with 1 point assigned to the presence of the initial code and 0 points assigned to the absence of the initial code. The item score was the sum of the scores of the initial codes under the item, and the domain score was the sum of the item scores under the domain. The total score was the sum of the three domain scores. According to the assignment rules, the matrix coding table in NVivo 12 Plus software was exported for statistical analysis.

The score rate (%) was calculated using Microsoft Office Excel 2019 software. Item score = actual total item score/theoretical maximum item score. Domain score rate = actual domain score/theoretical maximum domain score. Total score rate = total theoretical maximum score.

Statistically significant differences in the scoring rates of the subgroups under different basic feature classifications indicate that the TCM GPR methodology model has discriminability and is capable of distinguishing between different types of TCM guidelines.

##### 2.3.3.2 Subgroup analysis

Statistical analysis of subgroup scores was conducted based on the classification of basic features such as publication year, literature sources, development organizations, guideline types, discipline categories, and funding categories.

## 3 Results

### 3.1 Systematically identify research samples

**FIGURE 1:**
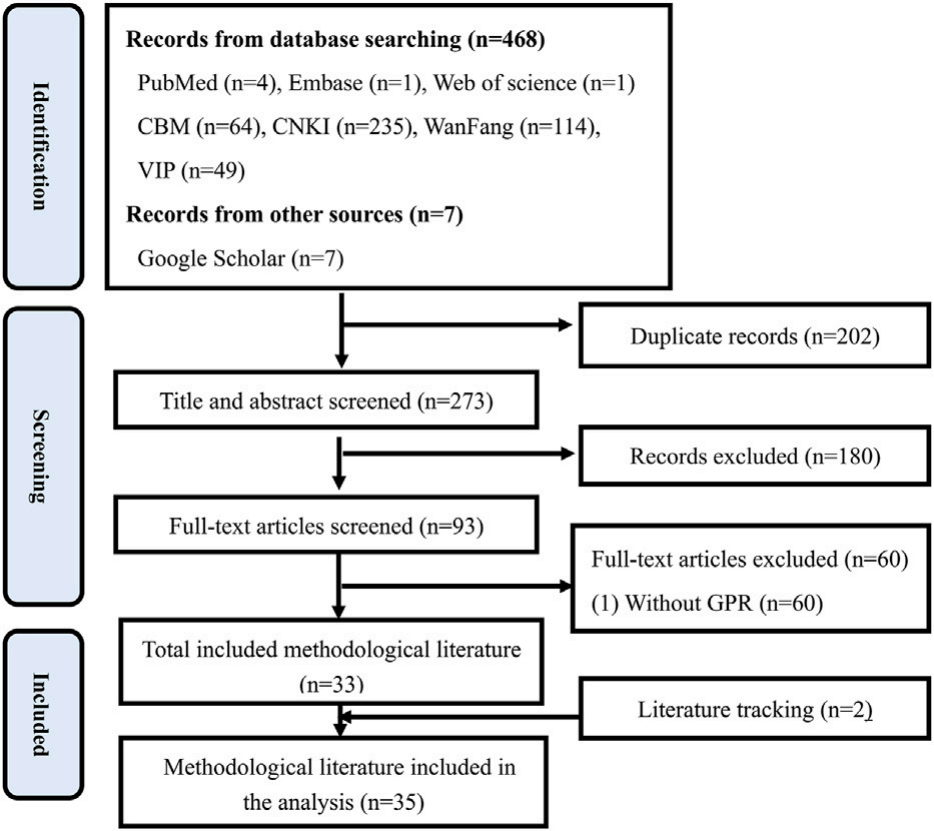
PRISMA flowchart for methodological literature.

**FIGURE 2:**
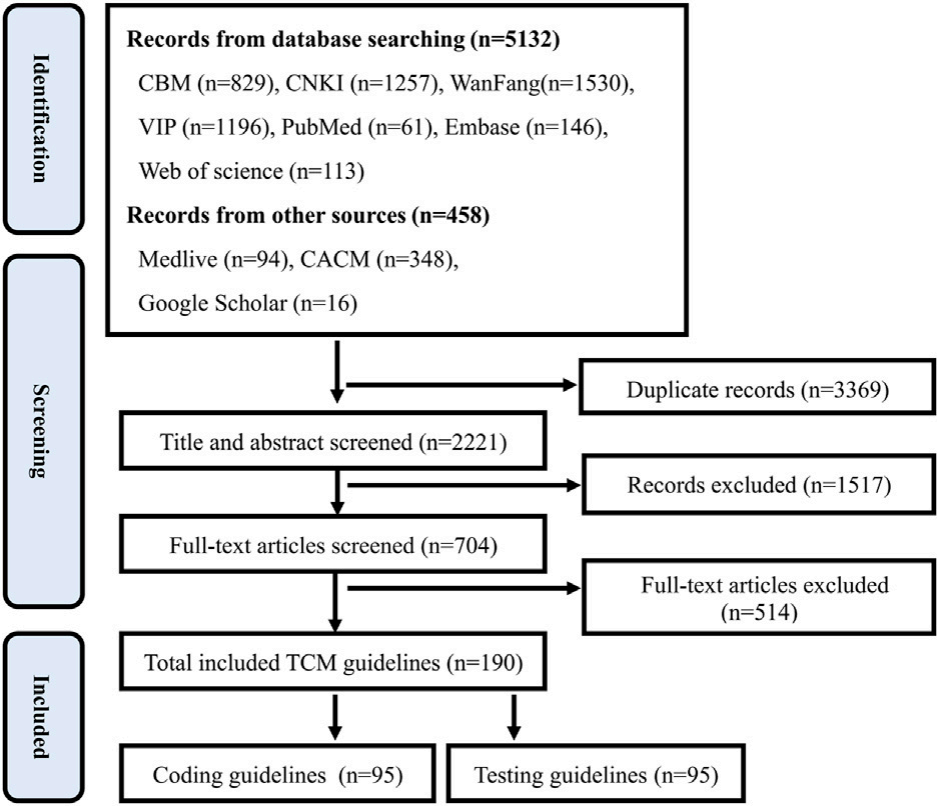
PRISMA flowchart for TCM guidelines.

A total of 35 methodological literature and 190 TCM guidelines were included. *Preferred Reporting Items for Systematic Reviews and Meta Analyses* (PRISMA) Flowchart (Page et al., 2021) for methodological literature and TCM guidelines was shown in Figures 1, 2.

The first methodological literature about TCM GPR was published in 2008. Since then, the annual average number of publications has stayed low but stable. 68.57% (24/35) of the methodological literature received funding support. The number of funded articles has shown a general upward trend.

The first TCM guidelines that mentioned the TCM GPR method were published in 2011, and the number of publications has generally been on the rise. Among the 190 guidelines, 60.00% (114/190) received funding support.

### 3.2 Model development

#### 3.2.1 Thematic analysis to generate an a priori framework

Through the logical framework analysis, a problem tree containing three key issues (unclear definitions, non-standardized procedures and methods, and non-uniformity of reporting forms) and nine phenomenon issues (role in terms of significance, conditions of use, scope of application, supporting information, procedures, methods, quality assessment, reporting content, and reporting presentation) was constructed. This was transformed into an initial analytical framework based on causal relationships (themes including definitions, procedures and methods, and reporting guideline, and sub-themes including role in terms of meaning, conditions of use, scope of application, supporting information, procedures, methods, quality assessment methods, reporting content, and reporting presentation).

Through thematic analysis, *a priori* framework comprising three main themes and ten sub-themes was extracted from the literature, as summarized in Table 1.

**TABLE 1:** A priori framework derived from thematic analysis.

Theme	Sub-themes	Categories
1 TCM-GPR definition	1.1 Major types	1.1.1 TCM GPR based on consensus
		1.1.2 TCM GPR based on good practice points
	1.2 Role and significance	1.2.1 As a supplement to evidence-based recommendations
		1.2.2 Manifesting characteristics of TCM clinical practice
		1.2.3 Facilitating the clinical application of TCM
		1.2.4 Providing research entry points for evidence-based Chinese medicine
	1.3 Conditions of use	1.3.1 Evidence condition
		1.3.2 Clinical question features
	1.4 Scope of application	1.4.1 TCM diagnosis
		1.4.2 TCM treatments
	1.5 Supporting information	1.5.1 Expert clinical experience and opinion
		1.5.2 Normative documents
		1.5.3 TCM indirect evidence
		1.5.4 TCM qualitative materials
2 TCM GPR procedure and methods	2.1 Development procedure	2.1.1 Defining the topic and scope
		2.1.2 Constructing groups
		2.1.3 Formulating clinical questions
		2.1.4 Determining whether or not to develop TCM GPR
		2.1.5 Collect information relevant to the questions
		2.1.6 Integrating relevant information
		2.1.7 Preparing the draft
		2.1.8 Reaching consensus
		2.1.9 Completing the final draft
		2.1.10 Consulting stakeholders
		2.1.11 Getting approval
	2.2 Development methods	2.2.1 Information collection methods
		2.2.2 Information integration methods
		2.2.3 Methods for going from integrated information to TCM GPR
	2.3 Quality evaluation methods	2.3.1 Quality evaluation of experts’ experience based on TCM ancient literature
		2.3.2 Quality evaluation of Personal experience of TCM experts
		2.3.3 Quality evaluation of experts’ experience based on modern TCM literature
3 TCM GPR reporting guideline	3.1 Reporting content	3.1.1 Title of TCM GPR
		3.1.2 Clear and specific TCM GPR
		3.1.3 Rationale for the GPR
	3.2 Features of the reporting format	3.2.1 Facilitating identification

**FIGURE 3:**
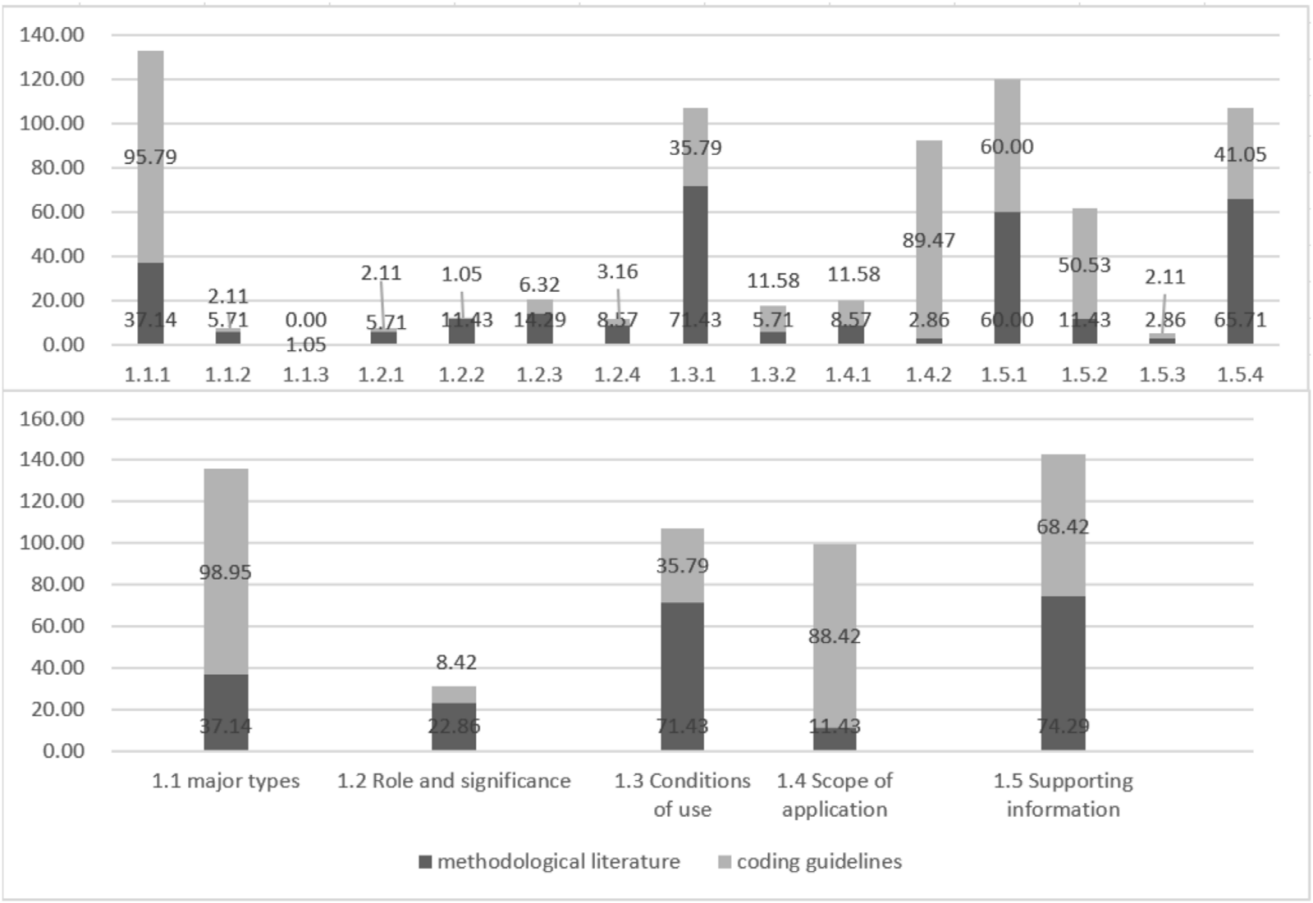
Coding support of methodological literature and coding guidelines for themes at all levels of TCM GPR definition.

Definition refers to what the TCM GPR is. 97.14% (34/35) of the methodological literature reported on this theme. Main Types describes the categories of methods integrated into the methodology model. Role and Significance describes the importance and significance of developing TCM GPR. Conditions of Use describes the conditions necessary for the development of TCM GPRs. Scope of Application describes the main areas where TCM GPRs can be developed. Supporting Information describes information that supports the formation of TCM GPR. The coding support of methodological literature for themes at all levels of TCM GPR Definition (the proportion of methodological literature mentioning the theme to the overall methodological literature) is detailed in Figure 3.

**FIGURE 4:**
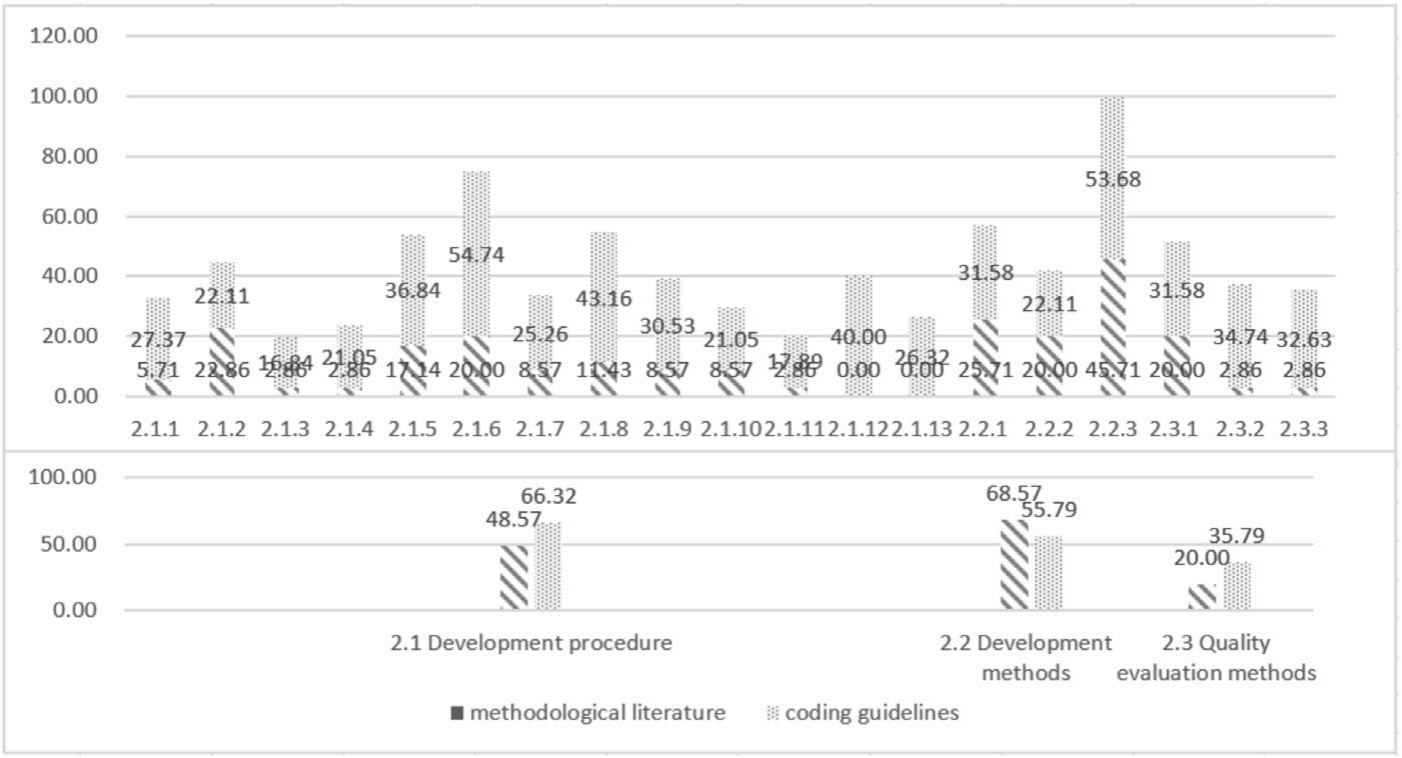
Coding support of methodological literature and coding guidelines for themes at all levels of TCM GPR procedures and methods.

Procedures and Methods refer to how to develop TCM GPR. 85.71% (30/35) of the methodological literature reported on this theme. Procedures describe the specific steps involved in developing the TCM GPR in chronological order. Methods describe the technical methods involved in developing the TCM GPR, and the quality assessment methodology describes the methods used to assess the quality of the supporting information, ensuring the credibility of the TCM GPR. The specific levels of support are shown in Figure 4.

**FIGURE 5:**
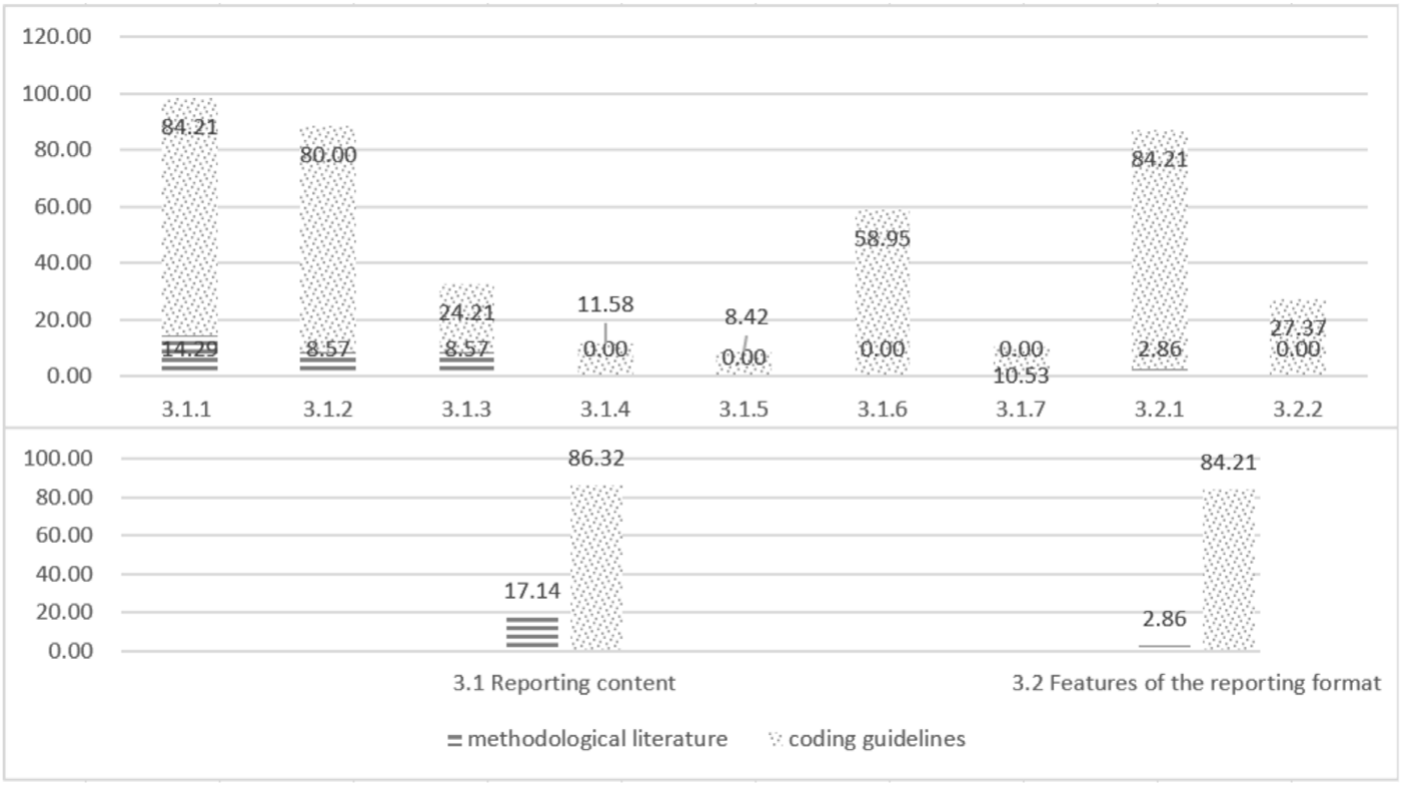
Coding support of methodological literature and coding guidelines for themes at all levels of TCM GPR reporting guideline.

Reporting guideline refers to how to report TCM GPR. It plays an important role in clearly expressing the content of TCM GPR and quickly identifying TCM GPR. 17.14% (6/35) of the methodological literature reported on this theme. Report Content describes the essential elements that should be included in the report. Features of the reporting format describe how the report should be presented for clarity and comprehensiveness. Specific levels of support are shown in Figure 5.

#### 3.2.2 Synthesizing the TCM GPR methodological model

The three main themes in the *a priori* framework were all supported by the TCM coding guidelines, with 100% (95/95), 78.95% (75/95), and 86.32% (82/95) levels of support respectively. All ten sub-themes were also supported by the coding guidelines. Specific levels of support are shown in Figures 3–5.

What could not be coded into the *a priori* framework was thematically analyzed to form new themes (sub-themes/categories). Themes and sub-themes remained unchanged, with eight new categories and four prior categories deepened. Ultimately, the *a priori* framework and new themes were merged to develop TCM GPR methodology model, including 3 themes, 10 sub-themes, and 42 categories, as shown in Table 2.

**TABLE 2:** Overall structure of themes (sub-themes/categories) and related definition.

Categories	Definitions
1.1.1 TCM GPR based on consensus	Other approaches to forming recommendations based on consensus when there is limited or no evidence are collectively referred to as consensus-based TCM GPR.
1.1.2 TCM GPR based on good practice points	Good Practice Points-based TCM GPRs are intended to assist guideline users by providing short pieces of recommendation which may not have an evidence base, but which are seen as essential to good clinical practice
*1.1.3 TCM GPR based on good practice statements*	*Good Practice statements-based TCM GPRs represent recommendations that guideline panels feel are important but that, in the judgment of GRADE working group, are not appropriate for formal ratings of quality of evidence. GPS should be developed when panels have high confidence that indirect evidence undoubtedly supports the net benefit and when, in addition, it would be an onerous and unproductive exercise and poor use of the panels’ limited resources to collect the evidence*
1.2.1 As a supplement to evidence-based recommendations	Role and Significance describes the importance and significance of TCM GPR. TCM GPR is 1) a type of recommendation when there is no evidence-based recommendation in the guideline, and it serves as a supplement to the evidence-based recommendations. TCM GPR retains the unique clinical characteristics of clinical practitioners of TCM, 2) manifests characteristics of TCM clinical practice, facilitates 3) the clinical application of TCM, and 4) provides a research entry point for the guidelines
1.2.2 Manifesting characteristics of TCM clinical practice	
1.2.3 Facilitating the clinical application of TCM	
1.2.4 Providing research entry points for evidence-based Chinese medicine	
1.3.1 Evidence condition	In the process of CPG development, a systematic search of relevant evidence for clinical questions is an important step. Evidence condition is subdivided into two subcategories: if there is no directly available evidence, or, if low-quality evidence contradicts the guideline panels’ perception of clinical practice, TCM GPR can be formed
1.3.2 Clinical question features	Clinical questions that require the formation of a TCM GPR should be 1) of high concern and wide-ranging in clinical practice, and *2) important and in urgent need of solution programs*
1.4.1 TCM diagnosis	In the field of TCM diagnosis, 1) Cause of disease, Mechanism of disease and 2) TCM pattern identification are the main focuses of developing TCM GPRs
1.4.2 TCM treatments	In the field of TCM treatment, 1) treatment of pattern identification, 2) symptomatic treatment, 3) preventive rehabilitation and regimen, *4) adverse effects of treatment, and 5) implementation and application of treatment* are the main focuses of the development of TCM GPR.
1.5.1 Expert clinical experience and opinion	Expert experience includes 1) expert experience based on TCM ancient literature; 2) expert experience based on modern TCM literature, such as medical cases, case reports, famous prescriptions, and other literature-type expert experience
1.5.2 Normative documents	Normative documents mainly include 1) TCM-related documents issued by the government, such as the Chinese Pharmacopoeia, the National Basic Drug List, the National Basic Medical Insurance Drug List, government reports, etc.,; 2) TCM laws and regulations, such as the latest regulatory documents; 3) the current TCM standards and norms, such as the planning textbooks, theoretical monographs, the latest extant guidelines and standardsetc.
1.5.3 TCM indirect evidence	When there is no evidence that directly proves the effect of the content proposed in the recommendation, it can be linked with other evidence to support the recommendation jointly
1.5.4 TCM qualitative materials	TCM qualitative research mainly includes information obtained by non-quantitative means such as qualitative research literature, expert opinions or assertions derived through consensus methods such as Delphi
2.1.1 Defining the topic and scope	The main content covered by the TCM guidelines and their scope
2.1.2 Constructing groups	According to the needs, the groups should be constructed, including consensus groups, steering committees, working groups, and external review groups. At the same time, the conflict of interest of the group members and the academic schools of TCM should be taken into consideration (as there are differences in the characteristics of medication and diagnostic and therapeutic habits of different academic schools, and the imbalance of experts from academic schools may have an impact on the conflict of non-financial interests as well as the achievement of consensus, etc.)
2.1.3 Formulating clinical questions	Develop issues of interest, incorporate them into the prioritization process, and identify specifics such as populations, interventions, comparators and outcome
2.1.4 Determining whether or not to develop TCM GPR	Determine the need for a TCM GPR based on the conditions of use and scope of application
2.1.5 Collecting relevant information	Collect supporting information for the TCM GPR, including expert experience, normative documents, indirect evidence of TCM and qualitative studies of TCM as mentioned in 1.5
2.1.6 Integrating relevant information	Adopt appropriate methods (i.e., the information integration methods mentioned in 2.2.2) to integrate the collected information and provide reference materials for subsequent consensus
2.1.7 Preparing the draft	Prepare a first draft based on the integrated supporting information with reference to the reporting guideline
2.1.8 Reaching consensus	On the basis of the first draft, invite experts to reach a consensus
2.1.9 Completing the final draft	Based on the results of the consensus, refine the first draft to form the final draft
2.1.10 Consulting stakeholders	Invite stakeholders to review the final draft to improve it
2.1.11 Getting approval	Submit the final draft to relevant organizations or institutions for review and approval
*2.1.12 Publishing and disseminating*	*The approved document is published, disseminated and promoted through existing channels*
*2.1.13 Assessing the need for update*	*Periodic reviews are conducted and if new evidence emerges, evidence-based, evidence-based recommendations should be developed*
2.2.1 Information collection methods	Describes how to comprehensively gather the information needed to develop the TCM GPR. Methods used to gather supporting information include 1) systematic TCM literature collection and 2) survey
2.2.2 Information integration methods	Describes the methods used to integrate the collected supporting information on TCM during the development of the TCM GPR. Methods used to integrate the collected information included 1) qualitative research and 2) qualitative evidence synthesis
2.2.3 Methods for going from integrated information to TCM GPR	Methods used to form recommendations based on the synthesized information when reaching consensus include 1) consensus methods (Delphi, modified Delphi, consensus conference, nominal group); 2) consensus rules; and 3) consensus considerations (*large and clear net benefits*, values and preferences, acceptability, *cost considerations, safety, applicability*)
2.3.1 Quality evaluation of experts’ experience based on TCM ancient literature	Methods for evaluating the quality of ancient literature
2.3.2 Quality evaluation of Personal experience of TCM experts	Methods for evaluating the empirical views of traditional Chinese medicine practitioners with profound medical knowledge and rich clinical experience
2.3.3 Quality evaluation of experts’ experience based on modern TCM literature	Methods for evaluating the quality of expert experience based on modern literature
3.1.1 Title of TCM GPR	Title used to signal TCM GPR.
3.1.2 Clear and specific TCM GPR	Presentation of recommendations used to provide specific practice guidance
3.1.3 Rationale for the GPR	Rationale for specific recommendations including supporting information for the recommendation and consensus results
*3.1.4 Clinical question*	*Report presentation of specific clinical questions*
*3.1.5 degree of consensus*	*Identifies the level of agreement with the consensus*
*3.1.6 strength of recommendations*	*Identifies the strength of the recommendation*
*3.1.7 Remarks*	*Includes issues such as conditions of use, dosage, and safety of the drug in the recommendation*
3.2.1 Facilitating identification	Identifiers and specific presentation formats for TCM GPRs so that they can be quickly found in the guideline
*3.2.2 Practicability*	*Specific recommendations are easy to understand and the content is actionable*

Italicized: new categories; “_”: deepened categories.

**FIGURE 6:**
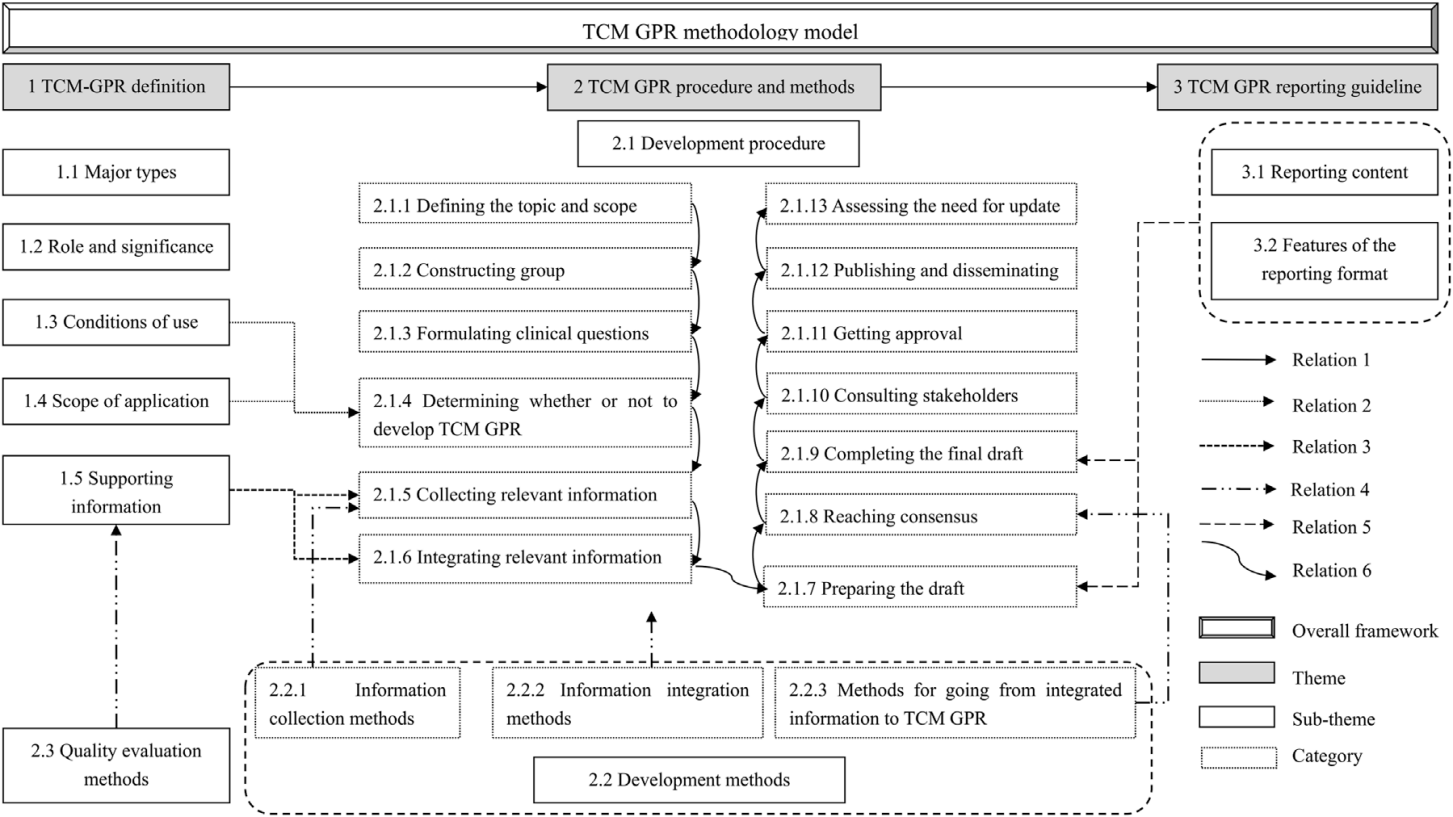
Thematic relations of TCM GPR methodology model.

The relations between themes, sub-themes, and categories are presented in a thematic relationship diagram, as shown in Figure 6. The relationships are as follows:

Relation 1: Definition, procedures and methods, and reporting guideline are the main themes of the TCM GPR methodology model. The need for a TCM GPR is determined based on its definition. Once established, the GPR is developed and reported according to the specified procedures and methods, and reporting guideline.

Relation 2: “Conditions of Use” and “Scope of Application” are key determinants for the applicability of a TCM GPR. When both conditions are met, a TCM GPR can be developed.

Relation 3: The information collected and integrated during the “Collecting relevant information” and “Integrating relevant information” steps is provided by the “Supporting information”.

Relation 4: In the “Collecting relevant information” step, information is gathered using specified “Information collection methods”. During the “Integrating relevantinformation” step, the collected information is synthesized using “Information integration methods”. Concurrently, a quality assessment method is employed to evaluate the collected supporting information. The “Methods for going from integrated information to TCM GPR” provides methodological support for the “Reaching consensus” step.

Relation 5: At the steps of the “preparing the draft” and the “Completing the final draft”, TCM GPR could be reported according to the “Reporting guideline”.

Relation 6: The sequence of development steps.

#### 3.2.3 Dissonance exploration

Compared with the *a priori* framework, the themes and sub-themes of the final TCM GPR methodology model remained unchanged, with eight new categories and four existing categories being further refined. The details are shown in Table 2.

### 3.3 Model validation

#### 3.3.1 Saturation test

None of the test guidelines indicated any newly emerging themes or sub-themes, leading to the conclusion that the study had reached theme saturation. See Supplementary Material 2 for details.

#### 3.3.2 Structural rationality test

TCM GPR methodology model includes 10 observational variables and requires at least 50 samples (Naidoo and Fields, 2019). With 190 TCM guidelines included in this study, the sample size is considered sufficient.

##### 3.3.2.1 Reliability

The Cronbach’s α of the 3 themes were 0.772, 0.574, and 0.654 in that order. As shown in Table 3.

**TABLE 3:** Results of the internal reliability analysis.

Theme	Cronbach′s α	Number of sub-themes	Internal consistency
1. TCM-GPR definition	0.772	5	Good
2. TCM GPR procedure and methods	0.574	3	Acceptable
3. TCM GPR reporting guideline	0.654	2	Good

##### 3.3.2.2 Validity

**FIGURE 7:**
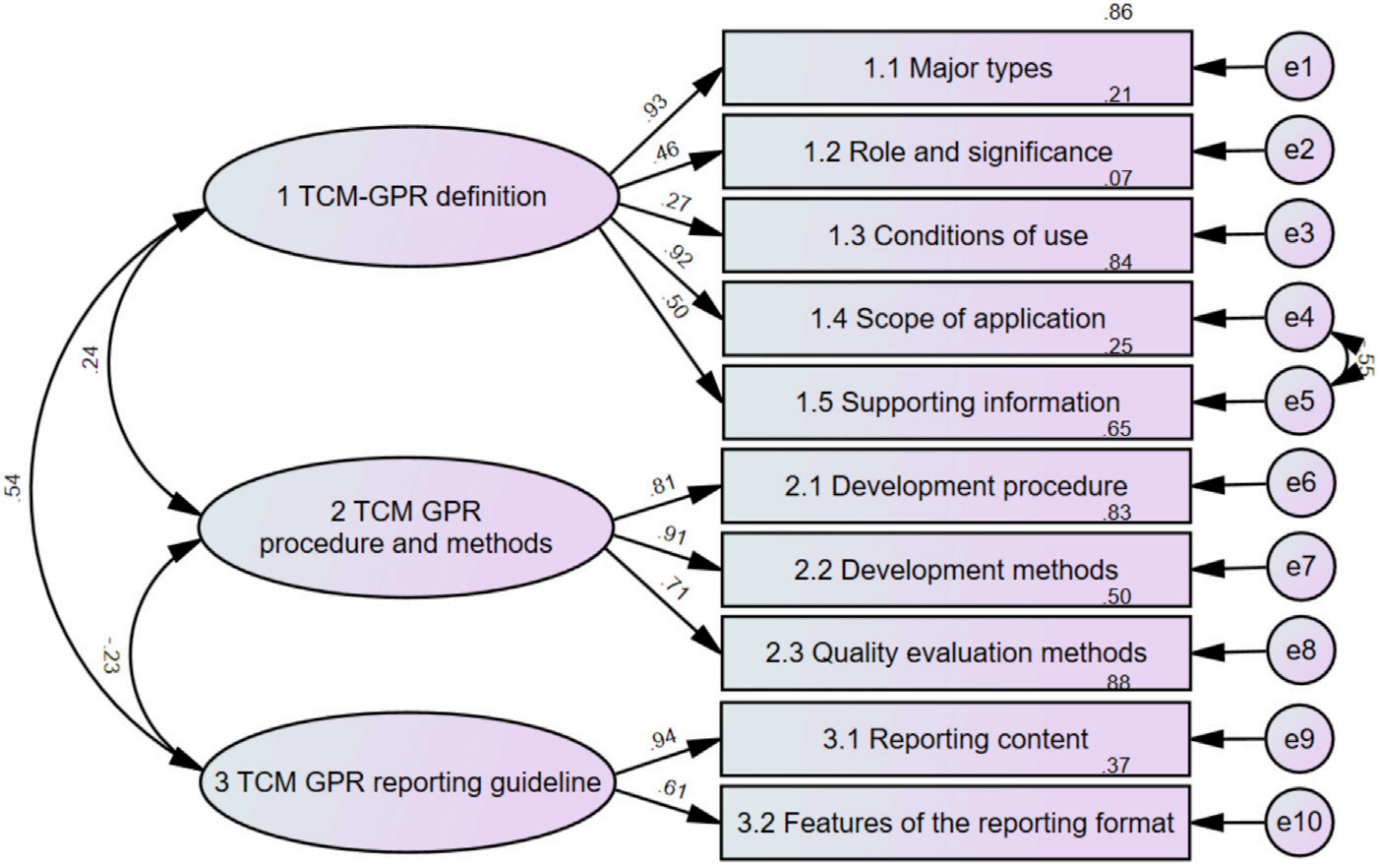
TCM GPR methodology model and path coefficients.

According to the data characteristics, a validation factor analysis was conducted by selecting Asymptotically distribution-free using the Analysis Properties column (Analysis Properties) in AMOS 21.0 software.The results are shown in Table 4: χ^2^/df was 2.988 (<3); the RMSEA was 0.103 (<0.11), indicating acceptable fit (Warlick et al., 2018); GFI was 0.932 (>0.9); and AGFI was 0.860 (>0.8). As shown in Figure 7, the standardized factor loadings of the subthemes were all >0.4 except for the (1.3) conditions of use.The CRs of the latent variables were 0.775, 0.852, and 0.762, which were all greater than 0.6 in that order, and the AVEs were 0.449, 0.660, and 0.662, in that order, except for “1 Definition of GPR in TCM”, which was greater than 0.5, See Table 5 for details.

**TABLE 4:** Confirmatory factor analysis results for the model.

Evaluation index	Evaluation criteria	Fit results
χ^2^/df	1.000–3.000 indicates good fit	2.988
GFI	>0.900, the closer to 1.000 the better	0.932
AGFI	>0.800, the closer to 1.000 the better	0.860
RMSEA	<0.100 is considered acceptable	0.103

χ^2^/df = chi-square/degrees of freedom ratio; GFI, goodness of fit index; AGFI, adjusted goodness of fit index; RMSEA, root mean square error of approximation.

**TABLE 5:** Results of the convergent validity of the model.

Latent variables	Observed variables	Standardized factor loadings	CR	AVE
1 TCM-GPR definition	1.1 Major types	0.928	0.775	0.449
	1.2 Role and significance	0.463		
	1.3 Formulation conditions	0.273		
	1.4 Scope of application	0.917		
	1.5 Supporting information	0.502		
2 TCM GPR procedure and methods	2.1 Development procedure	0.806	0.852	0.660
	2.2 Development methods	0.911		
	2.3 Quality assessment methods	0.708		
3 TCM GPR reporting guideline	3.1 Reporting content	0.938	0.762	0.626
	3.2 Features of the reporting format	0.610		

#### 3.3.3 Discriminability test

The results showed statistically significant differences in the total score rates of guidelines published by different literature sources, different development organizations, different guideline types, different discipline categories, and different funding categories. Statistically significant differences in the scoring rates of guidelines published in different years, different literature sources, and different development organizations on the definition, procedures and methods, and reporting guideline of TCM GPR, statistically significant differences in the scoring rates of guidelines published in different guideline types on the definition and reporting guideline of TCM GPR, and statistically significant differences in the scoring rates of guidelines published in different disciplinary categories, and different funding categories on the definition, procedures and methods, and reporting guideline of TCM GPR. As shown in Table 6.

**TABLE 6:** Discriminability test table.

Basic feature	Total	1	2	3
Publication year				
1.2023 (n = 24)	20.15	16.67	16.67^e^	42.59
2.2022 (n = 34)	23.84	21.93^e^	18.30	50.65^e^
3.2021 (n = 21)	25.02	24.68^e^	18.12	53.44^d,e^
4.2020 (n = 18)	15.67	15.91	11.27^e^	32.72c
5.2019 and before (n = 93)	23.01	13.15^b,c^	25.99^a,d^	35.24^b,c^
*F-value*	1.929	7.302	3.214	6.996
*P-value*	0.107	<0.001*	0.014*	<0.001*
Literature sources				
1.CJITWM(n = 24)	27.24^b,e^	26.14^b,d,e^	19.79^b^	59.72^b,c,e^
2.JPTCM(n = 23)	40.62^a,c,d,e^	14.62^a^	56.64^a,c,d,e^	40.10^a^
3.JTCM(n = 15)	22.29^b^	20.91^d,e^	19.26^b^	37.78^a^
4.CJTCMP(n = 10)	16.72^b^	12.27^a,c^	11.67^b^	47.78
5.Other (n = 118)	18.24^a,b^	15.02^a,c^	15.58^b^	36.72^a^
*F-value*	29.319	7.176	37.432	7.304
*P-value*	<0.001*	<0.001*	<0.001*	<0.001*
Development organizations	Total	1	2	3
1.CACM(n = 70)	16.27^b,d,e^	13.18^b,c^	13.02^d,e^	36.83^b^
2.CATCM(n = 22)	28.97^a,d^	27.89^a,d,e^	21.46^d^	61.62^a,d,e^
3.WFCMS(n = 14)	21.75^d^	26.95^a,d,e^	12.50^d,e^	46.03
4.AHNUCM(n = 9)	37.81^a,c,e^	14.65^b,c^	50.62^a,b,c,e^	43.21^b^
5.Other (n = 75)	24.28^a,d^	15.03^b,c^	26.78^a,c,d^	36.89^b^
*F-value*	11.737	14.344	12.378	7.835
*P-value*	<0.001*	<0.001*	<0.001*	<0.001*
Guideline types				
1.Traditional Chinese medicine (n = 138)	21.05^c^	14.59^c^	21.14	36.47^c,d^
2.Chinese and Western medicine (n = 22)	24.15	16.94^c^	24.12	41.92^c^
3.Proprietary Chinese Medicine (n = 22)	28.97^a^	27.90^a,b^	21.84	60.10^a,b^
4.Acupuncture and massage (n = 8)	21.08	21.59	11.81	56.94^a^
*F-value*	3.675	8.026	0.753	11.559
*P-value*	0.025*	0.001*	0.522	<0.001*
Discipline categories				
1.Internal medicine (n = 61)	19.45^b^	18.41	14.75^a,b^	40.80
2.Pediatrics (n = 34)	35.95^a,c,d,e^	17.38	44.28^a,c,d,e^	48.04
3.Orthopedics (n = 27)	17.36^b^	14.14	15.95^b^	30.86^a,b,d^
4.Obstetrics and Gynecology (n = 16)	25.09^b,c,d^	21.02	21.01^b^	51.39^e^
5.Other (n = 52)	18.51^b,d^	14.24	16.35^b^	37.61^d^
*F-value*	14.891	2.402	19.904	4.160
*P-value*	<0.001*	0.058	<0.001*	0.003*
Funding categories				
1. National level (n = 96)	26.35^e^	16.76	28.53^c,d,e^	41.09^d^
2.Provincial and ministerial level (n = 7)	18.76	16.23	17.46	30.16^c^
3.Academic (Association) (n = 7)	17.70	21.43	6.75^a^	52.38^b,d^
4.Departmental level (n = 4)	10.07	10.23	9.72^a^	11.11^a,c,e^
5.Not reported (n = 76)	18.64^a^	16.57	14.14^a^	41.67^d^
*F-value*	5.921	1.232	7.951	3.208
*P-value*	0.006*	0.345	<0.001*	0.014*

n, number of guidelines. *: statistically significant difference. a, b, c, d, e: denote statistically significant differences when compared with groups 1, 2, 3, 4, and 5, respectively. CJITWM: Chinese Journal of Integrated Traditional and Western Medicine; JPTCM, Journal of Pediatrics of Traditional Chinese Medicine. JTCM, Journal of Traditional Chinese Medicine; CJTCMP, China Journal of Traditional Chinese Medicine and Pharmacy; CACM: China Association of Chinese Medicine; CATCM: China Association of Traditional Chinese Medicine; WFCMS, World Federation of Chinese Medicine Societies; AHNUCM, affiliated hospital of nanjing university of chinese medicine.

## 4 Discussion

### 4.1 Increasing attention to TCM-GPR standardization

A total of 35 methodological papers and 190 TCM guidelines were analyzed in this study. Both showed an increasing trend in numbers, partly due to enhanced financial support, with most funded projects receiving national-level funding. This indicates an increasing emphasis on TCM-GPR standardization.

### 4.2 Model development

#### 4.2.1 Definition of TCM GPR

According to the “TCM GPR Definition”, the concept of TCM GPR can be outlined as follows: When clinical evidence is of low (or very low) quality and it is inconsistent with the experience of clinical practice of TCM, or no direct evidence is in place, TCM guidelines are needed for important and urgent questions that are of high concern and wide-ranging in clinical practice. Recommendations that reflect the characteristics of clinical practice of TCM can be formed based on expert clinical opinion and experience, normative documents, indirect evidence or qualitative research of TCM to guide and facilitate application of TCM treatments. Such recommendations are named TCM GPR.

#### 4.2.2 Quality evaluation methods

Expert experience is a crucial component in formulating TCM guidelines, necessitating a comprehensive evaluation of its reliability, authority, completeness, and source. Various methods for evaluating these aspects are mentioned in the collected literature. However, these methods typically serve as evidence-quality grading methods, and since expert experience is not derived from clinical research, these grading methods should not be directly applied. Instead, they can be used as quality grading methods for the literature to better guide clinical decision-making for TCM practitioners.

#### 4.2.3 Dissonance exploration

The Dissonance Exploration highlights the differences between the *a priori* framework and TCM GPR methodology model. These differences are categorized into two main scenarios: (i) themes (including sub-themes and categories) in the *a priori* framework that are not supported by coding in the TCM guidelines, and (ii) new themes (including sub-themes and sub-sub-themes) developed based on the coding guideline. The first scenario suggests that the theoretical approaches related to TCM GPR mentioned in methodological literature have not been systematically applied in practice. The second scenario indicates the limitations of these theoretical approaches. Both scenarios reflect a disconnection between theory and practice, underscoring the need for a TCM GPR methodology model.

Compared to the *a priori* framework, eight new sub-themes have been integrated into the TCM GPR methodology model, including five additional reporting guideline. While the methodological literature primarily focuses on the specific formulation of TCM GPR, the guidelines emphasize the standardization and normalization of reporting. Therefore, the TCM guidelines include increasingly detailed specifications for TCM GPR reporting and their presentation.

### 4.3 Model validation

#### 4.3.1 Saturation test

Data saturation is commonly employed to assess the adequacy of research data in qualitative studies and serves as a critical criterion for evaluating article quality (Yang et al., 2022; Constantinou et al., 2017). In this study, the themes of the TCM GPR methodology model reached saturation. This indicates that the research data was sufficient and validates the rigor and qualitative robustness of the model development.

#### 4.3.2 Structural rationality test

Confirmatory factor analysis was used to fit the real-world data from published TCM guidelines. The overall fit of the corrected final methodology model fell within an acceptable range, indicating a well-developed model.

This TCM GPR methodology model incorporates three themes as latent variables. The low intrinsic reliability of “TCM GPR procedures and methods” may stem from categorizing information from the same reference into different sub-themes. For instance, a reference using a particular method might be categorized under both “Development methods” and “Development procedures”, affecting intrinsic reliability. Additionally, the factor loading of “Conditions of Use” was below 0.4, possibly due to limited data in this sub-theme. At the same time, the AVE of “1 TCM GPR definition” was poor, probably due to the low factor loading of “conditions of use”.

Additionally, the weights of the three themes were calculated based on the standardized factor loadings between each theme and its subthemes, using the absolute value method. The specific approach involved summing the absolute values of the standardized factor loadings for all subthemes within each theme, then calculating the ratio of each theme’s total factor loading to the total factor loadings of all themes, which was used as the theme’s weight. The results showed that the weight of Theme 1 was 43.63%, the weight of Theme 2 was 34.43%, and the weight of Theme 3 was 21.96%. The theme weights reflect the relative importance or contribution of each theme within the overall model. Among the themes, the most important was “TCM GPR Definition,” followed by “TCM GPR Procedures and Methods,” and lastly “TCM GPR Reporting Guidelines.” The definition serves as the foundation for any practice recommendation and is the premise for all subsequent work. Without a clear definition, the formulation and reporting process will lose direction and consistency. Procedures and methods, on the other hand, are at the core of practice recommendations. Only through systematic procedures and scientific methods can the scientific and practical validity of the recommendations be ensured. Lastly, reporting guidelines are essential for the effective dissemination and implementation of the recommendations. Standardized reporting not only facilitates the execution of the recommendations but also provides crucial support for continuous improvement and monitoring evaluation.

#### 4.3.3 Discriminability test

The results of the discriminability test revealed significant statistical differences in the scoring rates of guideline subgroups based on different basic features. This indicates that the TCM GPR methodology model can effectively differentiate between various types of TCM guidelines, demonstrating its discriminability.

### 4.4 Shortcomings and prospects

This study employed a qualitative method to develop the TCM GPR methodology model, but qualitative methods are prone to subjective biases. To mitigate this, a mixed-methods approach combining qualitative and quantitative research was adopted, utilizing quantitative methods to validate the developed model and ensure its scientific rigor.

Currently, the TCM GPR methodology model provides a general theoretical framework for TCM GPR. However, it lacks specific and clear operational specifications. Future research should focus on developing a more detailed, specific, and operational TCM GPR methodology tool, integrating existing guideline methodology tools to guide the precise formulation and application of TCM GPR (Zhou et al., 2022; Xie et al., 2020).

## Data Availability

The original data presented in the study can be found in the article/Supplementary Material, further inquiries can be directed to the corresponding author.

## Abbreviations

TCM, traditional Chinese medicine; GPR, good practice recommendation; CACM, China Association of Chinese Medicine; RMSEA, root mean square error of approximation; GFI, goodness of fit index; AGFI, adjusted goodness of fit index; CR, composite reliability; AVE, average variance extracted.
