# Corrigendum: Development and validation a methodology model for traditional Chinese medicine good practice recommendation: an exploratory sequential mixed methods study

**DOI:** 10.3389/fphar.2025.1590371

**Published:** 2025-03-18

**Authors:** Su Li, Luan Zhang, Yangyang Wang, Runsheng Xie, Wenjia Chen, Myeong Soo Lee, Yasser Sami Amer, Amin Sharifan, Heba Hussein, Hui Li

**Affiliations:** ^1^ The Second Clinical College, Guangzhou University of Chinese Medicine, Guangzhou, Guangdong, China; ^2^ Guangzhou University of Chinese Medicine, Guangzhou, Guangdong, China; ^3^ Guangdong Provincial Hospital of Chinese Medicine, Guangzhou, China; ^4^ The First Affiliated Hospital of Guangzhou University of Chinese Medicine, Guangzhou, Guangdong, China; ^5^ KM Science Research Division, Korea Institute of Oriental Medicine (KIOM), Daejeon, Republic of Korea; ^6^ Department of Pediatrics, King Saud University Medical City, Riyadh, Saudi Arabia; ^7^ Research Chair for Evidence-Based Health Care and Knowledge Translation, Family and Community Medicine Department, College of Medicine, King Saud University, Riyadh, Saudi Arabia; ^8^ Department of Internal Medicine, Ribeirão Preto Medical School, University of São Paulo, Ribeirão Preto, Brazil; ^9^ Department for Evidence-Based Medicine and Evaluation, University for Continuing Education Krems, Krems an der Donau, Lower Austria, Austria; ^10^ Oral Medicine, Oral Diagnosis, and Periodontology Department, Faculty of Dentistry, Cairo University, Cairo, Egypt; ^11^ State Key Laboratory of Traditional Chinese Medicine Syndrome, Research Group of Standardization of Chinese Medicine, The Second Affiliated Hospital of Guangzhou University of Chinese Medicine, Guangzhou, China

**Keywords:** traditional chinese medicine (TCM), guidelines, good practice recommendation (GPR), methodology model, mixed method

In the published article, there was an error in **Affiliations 1, 2, and 10**. Instead of “^1^Guangzhou University of Chinese Medicine, Guangzhou, Guangdong, China,” “^2^The Second Clinical College, Guangzhou University of Chinese Medicine, Guangzhou, Guangdong, China” and “^10^Oral Medicine, Oral Diagnosis, and Periodontology Department, Cairo University, Giza, Giza, Egypt,” it should be; *“* ^1^The Second Clinical College, Guangzhou University of Chinese Medicine, Guangzhou, Guangdong, China,” “^2^Guangzhou University of Chinese Medicine, Guangzhou, Guangdong, China,” and “^10^Oral Medicine, Oral Diagnosis, and Periodontology Department, Faculty of Dentistry, Cairo University, Cairo, Egypt.”

The authors apologize for this error and state that this does not change the scientific conclusions of the article in any way. The original article has been updated.

