# Supplementary material for: Development and validation a methodology model for traditional Chinese medicine good practice recommendation: an exploratory sequential mixed methods study
Source: Front Pharmacol. 2025 Jan 31;16:1501634. doi: 10.3389/fphar.2025.1501634 (PMC11825458; doi:10.3389/fphar.2025.1501634)
Supplement: Supplementary file 2 [file Table1.docx]

**Methodological literature search strategy**

**1 PubMed**

#1 ("Guidelines as Topic"[MeSH Major Topic]) OR (guideline*[Title]) OR (guidance*[Title]) OR (recommendation*[Title])

#2 "Practice Guideline" [Publication Type] OR "Guideline" [Publication Type]

#3 #1 OR #2

#4 (“medicine, Chinese Traditional”[MeSH Major Topic]) OR ("Traditional Chinese medicine"[Title/Abstract]) OR ("Chinese medicine"[Title/Abstract]) OR ("Chinese herbal"[Title/Abstract]) OR ("TCM"[Title/Abstract]) OR ("Acupuncture"[Title/Abstract]) OR ("Chinese massage"[Title/Abstract]) OR ("tuina"[Title/Abstract])

#5 #3 OR #4

#6 (((((((((((((((((((("lack of evidence"[Title/Abstract])) OR ("absence of evidence"[Title/Abstract])) OR ("indirect evidence"[Title/Abstract])) OR ("no evidence"[Title/Abstract])) OR ("lacking evidence"[Title/Abstract])) OR ("limited evidence"[Title/Abstract])) OR ("nonexistent evidence"[Title/Abstract])) OR ("insufficient evidence"[Title/Abstract])) OR ("poor evidence"[Title/Abstract])) OR ("little high quality evidence"[Title/Abstract])) OR ("low level of evidence"[Title/Abstract])) OR ("low quality evidence"[Title/Abstract])) OR ("expert opinion*"[Title/Abstract])) OR ("experts opinion*"[Title/Abstract])) OR ("extensive experience*"[Title/Abstract])) OR ("clinical expertise"[Title/Abstract])) OR ("experts experience*"[Title/Abstract])) OR ("expert experience*"[Title/Abstract])) OR ("clinical experience*"[Title/Abstract])) OR ("qualitative research"[Title/Abstract])

#7 (((("develop*"[Title]) OR ("handbook*"[Title])) OR (methodolog*[Title])) OR (manual*[Title])) OR (toolkit*[Title])

#8 #5 AND #6 AND #7 Filters: Full text

**2 Embase**

#1 guideline*:ti OR guidance*:ti OR recommendation*:ti

#2 'Traditional Chinese medicine':ti,ab OR 'Chinese medicine':ti,ab OR 'Chinese herbal':ti,ab OR 'TCM':ti,ab OR 'Acupuncture':ti,ab OR 'Chinese massage':ti,ab OR 'tuina':ti,ab

#3 #1 AND #2

#4 #3 AND [embase]/lim NOT ([embase]/lim AND [medline]/lim)

#5 #3 AND [embase]/lim NOT ([embase]/lim AND [medline]/lim) AND ('article'/it OR 'article in press'/it)

#6 'lack of evidence':ti,ab OR 'absence of evidence':ti,ab OR 'indirect evidence':ti,ab OR 'no evidence':ti,ab OR 'lacking evidence':ti,ab OR 'limited evidence':ti,ab OR 'nonexistent evidence':ti,ab OR 'insufficient evidence':ti,ab OR 'poor evidence':ti,ab OR 'little high quality evidence':ti,ab OR 'low level of evidence':ti,ab OR 'low quality evidence':ti,ab OR 'expert opinion*':ti,ab OR 'experts opinion*':ti,ab OR 'extensive experience*':ti,ab OR 'clinical expertise':ti,ab OR 'experts experience*':ti,ab OR 'expert experience*':ti,ab OR 'clinical experience*':ti,ab OR 'qualitative research':ti,ab OR '':ti,ab OR '':ti,ab OR '':ti,ab OR '':ti,ab OR '':ti,ab

#7 develop*:ti OR handbook*:ti OR methodolog*:ti OR manual*:ti OR toolkit*:ti

#8 #5 AND #6 AND #7

**3 Web of science**

#1 ((TI=(guideline*)) OR TI=(guidance*)) OR TI=(recommendation*)

#2 (((((((TS=("Traditional Chinese medicine")) OR TS=("Chinese medicine")) OR TS=("Chinese herbal")) OR TS=("TCM")) OR TS=("Acupuncture")) OR TS=("Chinese massage")) OR TS=("tuina"))

#3 #2 AND #1

#4 #2 AND #1 and MEDLINE® (数据库)

#5 #3 NOT #4

#6 ((((((((((((((((((((TS=("lack of evidence")) OR TS=("absence of evidence")) OR TS=("indirect evidence")) OR TS=("no evidence")) OR TS=("lacking evidence")) OR TS=("limited evidence")) OR TS=("nonexistent evidence")) OR TS=("insufficient evidence")) OR TS=("poor evidence")) OR TS=("little high quality evidence")) OR TS=("low level of evidence")) OR TS=("low quality evidence")) OR TS=("expert opinion*")) OR TS=("experts opinion*")) OR TS=("extensive experience*")) OR TS=("clinical expertise")) OR TS=("experts experience*")) OR TS=("expert experience*")) OR TS=("clinical experience*")) OR TS=("qualitative research"))

#7 ((((TI=("develop*")) OR TI=("handbook*")) OR TI=(methodolog*)) OR TI=(manual*)) OR TI=(toolkit*)

# 8 #5 AND #6 AND #7

**4 CNKI**

#1 TI='指南'+'指引'+'推荐意见'

#2 TI='中医'+'中药'+'中西医'+'中成药'+'针灸'+'针刺'+'推拿'+'传统医学'

#3 FT='非直接证据'+'无证据'+'证据不存在'+'没有证据'+'没有研究证据'+'低质量证据'+'低等级证据'+'高质量证据缺乏'+'证据有限'+'证据不充分'+'证据不足'+'缺乏证据'+'定性研究' +'质性研究'+'专家意见'+'专家经验'+'临床经验'+'共识'+'专家推荐'+'专家建议'

#4 TI='方法'+'手册'+'工具'+'构建'+'编制'+'制订'+'制定'+'研制'+'修订'

#5 #1 and #2 and #3 and #4

((TI='指南'+'指引'+'推荐意见') AND (TI='中医'+'中药'+'中西医'+'中成药'+'针灸'+'针刺'+'推拿'+'传统医学') AND (FT='非直接证据'+'无证据'+'证据不存在'+'没有证据'+'没有研究证据'+'低质量证据'+'低等级证据'+'高质量证据缺乏'+'证据有限'+'证据不充分'+'证据不足'+'缺乏证据'+'定性研究' +'质性研究'+'专家意见'+'专家经验'+'临床经验'+'共识'+'专家推荐'+'专家建议')) AND (TI='方法'+'手册'+'工具'+'构建'+'编制'+'制订'+'制定'+'研制'+'修订')

**5 Wangfang**

#1 题名:("指南" or ”指引” or ”推荐意见”)

#2 题名:(“中医” or ”中药” or ”中西医” or ”中成药” or ”针灸” or “针刺” or “推拿” or “传统医学”)

#3 全部:("非直接证据" or "无证据" or "证据不存在" or "没有证据"or "没有研究证据" or "低质量证据" or "低等级证据" or "高质量证据缺乏" or "证据有限" or "证据不充分"or "证据不足" or ”缺乏证据” or "定性研究" or ”质性研究” or "专家意见" or "专家经验" or "临床经验" or ”共识” or “专家推荐” or “专家建议”)

#4 题名:("方法" or "手册" or "工具" or "构建" or "编制" or "制定" or "制订" or "研制" or "修订")

#5 #1 and #2 and #3 and #4

(题名:("指南" or ”指引” or ”推荐意见”) and 题名:(“中医” or ”中药” or ”中西医” or ”中成药” or ”针灸” or “针刺” or “推拿” or “传统医学”) and 全部:("非直接证据" or "无证据" or "证据不存在" or "没有证据"or "没有研究证据" or "低质量证据" or "低等级证据" or "高质量证据缺乏" or "证据有限" or "证据不充分"or "证据不足" or ”缺乏证据” or "定性研究" or ”质性研究” or "专家意见" or "专家经验" or "临床经验" or ”共识” or “专家推荐” or “专家建议”)) and 题名:("方法" or "手册" or "工具" or "构建" or "编制" or "制定" or "制订" or "研制" or "修订")

**6 VIP**

#1 T=("指南" or ”指引” or ”推荐意见”)

#2 T=(“中医” or ”中药” or ”中西医” or ”中成药” or ”针灸” or “针刺” or “推拿” or “传统医学”)

#3 U=("非直接证据" or "无证据" or "证据不存在" or "没有证据"or "没有研究证据" or "低质量证据" or "低等级证据" or "高质量证据缺乏" or "证据有限" or "证据不充分"or "证据不足" or ”缺乏证据” or "定性研究" or ”质性研究” or "专家意见" or "专家经验" or "临床经验" or ”共识” or “专家推荐” or “专家建议”)

#4 T=("方法" or "手册" or "工具" or "构建" or "编制" or ”制定” or ”制订” or ”研制” or ”修订”)

#5 #1 and #2 and #3 and #4

(T=("指南" or ”指引” or ”推荐意见”) and T=(“中医” or ”中药” or ”中西医” or ”中成药” or ”针灸” or “针刺” or “推拿” or “传统医学”) and U=("非直接证据" or "无证据" or "证据不存在" or "没有证据"or "没有研究证据" or "低质量证据" or "低等级证据" or "高质量证据缺乏" or "证据有限" or "证据不充分"or "证据不足" or ”缺乏证据” or "定性研究" or ”质性研究” or "专家意见" or "专家经验" or "临床经验" or ”共识” or “专家推荐” or “专家建议”)) and T=("方法" or "手册" or "工具" or "构建" or "编制" or ”制定” or ”制订” or ”研制” or ”修订”)

**7 CBM**

#1 "指南"[中文标题:智能] OR "指南"[不加权:扩展] OR "指引"[中文标题:智能] OR "推荐意见"[中文标题:智能])

#2 "中医"[中文标题:智能] OR "中药"[中文标题:智能] OR "中西医"[中文标题:智能] OR "中成药"[中文标题:智能] OR "针灸"[中文标题:智能] OR "针刺"[中文标题:智能] OR "推拿"[中文标题:智能] OR "传统医学"[中文标题:智能]

#3 "非直接证据"[常用字段:智能] OR "无证据"[常用字段:智能] OR "证据不存在"[常用字段:智能] OR "没有证据"[常用字段:智能] OR "没有研究证据"[常用字段:智能] OR "低质量证据"[常用字段:智能] OR "低等级证据"[常用字段:智能] OR "高质量证据缺乏"[常用字段:智能] OR "证据有限"[常用字段:智能] OR "证据不充分"[常用字段:智能] OR "定性研究"[常用字段:智能] OR "质性研究"[常用字段:智能] OR "缺乏证据"[常用字段:智能] OR "共识"[常用字段:智能] OR "专家意见"[常用字段:智能] OR "专家经验"[常用字段:智能] OR "临床经验"[常用字段:智能] OR "专家推荐"[常用字段:智能] OR "专家建议"[常用字段:智能]

#4 "方法"[中文标题:智能] OR "工具"[中文标题:智能] OR "手册"[中文标题:智能] OR "构建"[中文标题:智能] OR "编制"[中文标题:智能] OR "制定"[中文标题:智能] OR "制订"[中文标题:智能] OR "研制"[中文标题:智能] OR "修订"[中文标题:智能]

#5 #1 AND #2 AND #3 AND #4

("指南"[中文标题:智能] OR "指南"[不加权:扩展] OR "指引"[中文标题:智能] OR "推荐意见"[中文标题:智能]) AND ("中医"[中文标题:智能] OR "中药"[中文标题:智能] OR "中西医"[中文标题:智能] OR "中成药"[中文标题:智能] OR "针灸"[中文标题:智能] OR "针刺"[中文标题:智能] OR "推拿"[中文标题:智能] OR "传统医学"[中文标题:智能]) AND ("非直接证据"[常用字段:智能] OR "无证据"[常用字段:智能] OR "证据不存在"[常用字段:智能] OR "没有证据"[常用字段:智能] OR "没有研究证据"[常用字段:智能] OR "低质量证据"[常用字段:智能] OR "低等级证据"[常用字段:智能] OR "高质量证据缺乏"[常用字段:智能] OR "证据有限"[常用字段:智能] OR "证据不充分"[常用字段:智能] OR "定性研究"[常用字段:智能] OR "质性研究"[常用字段:智能] OR "缺乏证据"[常用字段:智能] OR "共识"[常用字段:智能] OR "专家意见"[常用字段:智能] OR "专家经验"[常用字段:智能] OR "临床经验"[常用字段:智能] OR "专家推荐"[常用字段:智能] OR "专家建议"[常用字段:智能]) AND ("方法"[中文标题:智能] OR "工具"[中文标题:智能] OR "手册"[中文标题:智能] OR "构建"[中文标题:智能] OR "编制"[中文标题:智能] OR "制定"[中文标题:智能] OR "制订"[中文标题:智能] OR "研制"[中文标题:智能] OR "修订"[中文标题:智能])

**8 Gray literature——Google Scholar**

“tradition Chinese medicine guideline”、“Acupuncture”、“Chinese massage”

**TCM guideline search strategy**

**1 PubMed**

#1 ("Guidelines as Topic"[MeSH Major Topic]) OR (guideline*[Title]) OR (guidance*[Title]) OR (recommendation*[Title])

#2 "Practice Guideline" [Publication Type] OR "Guideline" [Publication Type]

#3 #1 OR #2

#4 (“medicine, Chinese Traditional”[MeSH Major Topic]) OR ("Traditional Chinese medicine"[Title/Abstract]) OR ("Chinese medicine"[Title/Abstract]) OR ("Chinese herbal"[Title/Abstract]) OR ("TCM"[Title/Abstract]) OR ("Acupuncture"[Title/Abstract]) OR ("Chinese massage"[Title/Abstract]) OR ("tuina"[Title/Abstract])

#5 #3 AND #4

**2 Embase**

#1 guideline*:ti OR guidance*:ti OR recommendation*:ti

#2 'Traditional Chinese medicine':ti,ab OR 'Chinese medicine':ti,ab OR 'Chinese herbal':ti,ab OR 'TCM':ti,ab OR 'Acupuncture':ti,ab OR 'Chinese massage':ti,ab OR 'tuina':ti,ab

#3 #1 AND #2

#4 #3 AND [embase]/lim NOT ([embase]/lim AND [medline]/lim)

#5 #3 AND [embase]/lim NOT ([embase]/lim AND [medline]/lim) AND ('article'/it OR 'article in press'/it)

**3 web of science**

#1 ((TI=(guideline*)) OR TI=(guidance*)) OR TI=(recommendation*)

#2 (((((((TS=("Traditional Chinese medicine")) OR TS=("Chinese medicine")) OR TS=("Chinese herbal")) OR TS=("TCM")) OR TS=("Acupuncture")) OR TS=("Chinese massage")) OR TS=("tuina"))

#3 #2 AND #1

#4 #2 AND #1 and MEDLINE® (数据库)

#5 #3 NOT #4

**4 CNKI**

#1 TI='指南'+'指引'+'推荐意见'

#2 TI='中医'+'中药'+'中西医'+'中成药'+'针灸'+'针刺'+'推拿'+'传统医学'

#3 #1 AND #2

(TI='指南'+'指引'+'推荐意见') AND (TI='中医'+'中药'+'中西医'+'中成药'+'针灸'+'针刺'+'推拿'+'传统医学')

**5 Wangfang**

#1 题名:("指南" or "指引" or "推荐意见")

#2 题名:("中医" or "中药" or "中西医" or "中成药" or "针灸" or "针刺" or "推拿" or "传统医学")

#3 #1 and #2

题名:("指南" or "指引" or "推荐意见") and 题名:("中医" or "中药" or "中西医" or "中成药" or "针灸" or "针刺" or "推拿" or "传统医学")

**6 VIP**

#1 T=("指南" OR "指引" OR "推荐意见")

#2 T=("中医" OR "中药" OR "中西医" OR "中成药" OR "针灸" OR "针刺" OR "推拿" OR "传统医学")

#3 #1 and #2

T=("指南" OR "指引" OR "推荐意见") and T=("中医" OR "中药" OR "中西医" OR "中成药" OR "针灸" OR "针刺" OR "推拿" OR "传统医学")

**7 CBM**

#1 ("指南"[中文标题:智能] OR "指南"[不加权:扩展] OR "指引"[中文标题:智能] OR "推荐意见"[中文标题:智能])

#2 ("中医"[中文标题:智能] OR "中药"[中文标题:智能] OR "中西医"[中文标题:智能] OR "中成药"[中文标题:智能] OR "针灸"[中文标题:智能] OR "针刺"[中文标题:智能] OR "推拿"[中文标题:智能] OR "传统医学"[中文标题:智能])

#3 #1 and #2

("指南"[中文标题:智能] OR "指南"[不加权:扩展] OR "指引"[中文标题:智能] OR "推荐意见"[中文标题:智能]) AND ("中医"[中文标题:智能] OR "中药"[中文标题:智能] OR "中西医"[中文标题:智能] OR "中成药"[中文标题:智能] OR "针灸"[中文标题:智能] OR "针刺"[中文标题:智能] OR "推拿"[中文标题:智能] OR "传统医学"[中文标题:智能])

**8 Medlive (94)**

检索词："中医指南" 、 "中药指南" 、 "中西医指南" 、 "中成药指南" 、 "针灸指南" 、 "针刺指南" 、 "推拿指南"

**9 China Association of Chinese Medicine**

Screening at the website of China Association of Chinese Medicine

**10 Gray literature ——Google Scholar**

“tradition Chinese medicine guideline”、“Acupuncture”、“Chinese massage”
